# Prevalence of Antimicrobial Resistance in Bacterial Isolates from Dogs and Cats in a Veterinary Diagnostic Laboratory in Colombia from 2016–2019

**DOI:** 10.3390/vetsci7040173

**Published:** 2020-11-10

**Authors:** David A. Gómez-Beltrán, David Villar, Sara López-Osorio, Duncan Ferguson, Laura K. Monsalve, Jenny J. Chaparro-Gutiérrez

**Affiliations:** 1 CIBAV Research Group, Facultad de Ciencias Agrarias, Universidad de Antioquia UdeA, Calle 70 No. 52-21, Medellín 050010, Colombia; david.gomezb@udea.edu.co (D.A.G.-B.); sara.lopezo@udea.edu.co (S.L.-O.); lkatherine.monsalve@udea.edu.co (L.K.M.); jenny.chaparro@udea.edu.co (J.J.C.-G.); 2 Department of Veterinary Biosciences, College of Veterinary Medicine, University of Illinois, Champaign, IL 61820, USA; dcf@illinois.edu

**Keywords:** antimicrobial resistance, multidrug resistance, dogs, cats, Colombia

## Abstract

The susceptibility to antimicrobials of bacterial isolates from dogs (*n* = 1256) and cats (*n* = 94) was retrospectively evaluated in a veterinary diagnostic laboratory over a 4-year period (2016–2019). Out of 1316 isolates in dogs, 771 were *Staphylococcus* spp. distributed as follows: *Staph. pseudointermedius* (*n* = 406), *Staph. intermedius* (*n* = 160), *Staph. aureus* (*n* = 104), and *Staph.* coagulase-negative (*n* = 101). In common, all *Staphylococcus* spp. showed a high prevalence (20–50%) of resistance to ampicillin, cephalosporin, enrofloxacin, gentamicin, tetracycline, and trimethoprim-sulfonamide, but a low prevalence (1–10%) of resistance to amoxicillin-clavulanate. With regards to the other families of bacteria, the number of antimicrobials for which resistance was high (>20%) in dogs was: *Enterobacteriaceae* (7/12), *Enterococcus* spp. (10/16), *E. coli* (11/15), *Pseudomonas* spp. (10/13), and *Streptococcus* spp. (4/9). For urinary tract infections caused by *E. coli* or *Enterobacteriaceae* (*Klebsiella* spp., *Proteus* spp.), amikacin and florfenicol were the only drugs that demonstrated 100% in vitro efficacy. Multi-drug resistance was observed in 18.7% (246/1316) and 22% (21/97) of the isolates from dogs and cats, respectively. Except for *Pseudomonas* spp., known for intrinsic resistance, resistance in other bacteria was likely attributed to high selection pressure. In conclusion, empirical antimicrobial use cannot be recommended to treat most common infections, and selection should be based on results from susceptibility testing.

## 1. Introduction

The emergence of multidrug-resistant bacteria in companion animals is an increasing concern as it narrows the potential use of antimicrobials for the treatment of infections. Because antimicrobial resistance (AMR) is constantly evolving, studies that monitor AMR regularly are important in order to guide therapeutic decisions and develop up-to-date control strategies. In general, variation in resistance complicates empirical selection of antimicrobial agents, and enhances the need for culturing and susceptibility testing. In addition, although the organisms in pets that most commonly cause disease tend to be different from humans, this is not always the case, and there is always the potential for the passage of drug-resistant genes and transmission between humans and pets.

Most studies in different countries investigate trends and/or patterns in resistance by focusing on a specific pathogenic bacterium (i.e., *Escherichia coli*) or a specific organ/system (i.e., urinary tract infections). However, a few studies from veterinary diagnostic laboratories have provided information on AMR patterns and trends in bacteria isolated from clinical samples submitted over the course of 10–20 years [1,2]. Unfortunately, in Colombia, the only coordinated program for antimicrobial resistance that operates in animals is occurring in the poultry sector, and includes monitoring at poultry farms, slaughterhouses and retail markets [3]. Because there have been no studies conducted in companion animals, the extent and importance of AMR in such veterinary practices remains unknown in Colombia. Thus, the objectives of this study were to estimate the prevalence of antimicrobial resistance of pathogens in dogs and cats with ear, skin, and urinary tract infections sampled at the College of Veterinary Medicine Teaching Hospital of Antioquia, Medellin. Data from this study could be used to guide veterinarian’s decisions and serve as a reference to start monitoring antimicrobial resistance throughout Colombia.

## 2. Materials and Methods

Clinical samples submitted for culture and susceptibility testing from cats and dogs from 2016 to 2019 were retrieved from the database of the Veterinary Diagnostic Laboratory of the University of Antioquia. Only complete records with no missing information of the samples were included in the study, and so, 66 cases were discarded, 60 from dogs and 6 from cats. The total number of complete records in dogs and cats was 1256 and 94, respectively. Blood agar plates were incubated with 5% CO_2_ while MacConkey agar plates were incubated aerobically. All samples were incubated at 37 °C for 18 to 24 h until adequate growth was present. Identification was based on colony type and morphology, Gram staining characteristics, and standard biochemical tests. Antimicrobiological susceptibility (AST) was undertaken using the Kirby-Bauer disk diffusion method [4]. Zones of growth inhibition were interpreted according to the Clinical and Laboratory Standard Institute (CLSI) guidelines [5]. Intermediate isolates were infrequent and regarded as resistant. The following genera of bacteria were included within the *Enterobacteriaceae* group: *Enterobacter*, *Klebsiella*, *Citrobacter*, *Proteus*, *Salmonella*, *Serratia*. *Escherichia* was considered separate from the *Enterobacteriaceae* group as it was the most prevalent uropathogen isolated. Within the *Pseudomonas* group, the following bacteria were included: *Pseudomonas*, *Flavimonas*, and *Acinetobacter*. 

The antimicrobials used to determine susceptibilities varied depending on specific requests by the veterinarian, but typically included: amikacin, amoxicillin-clavulanate, ampicillin, cephalothin, cephalosporin, enrofloxacin, gentamicin, trimethoprim-sulfonamide, doxycycline, tetracycline, ciprofloxacin, and florfenicol. The number of antimicrobials requested in dogs was larger than cats, some of the antimicrobials tested only in dogs were: penicillin, ampicillin-sulfactam, neomycin, chloramphenicol, and cefoperazone. Isolates showing resistance to three or more antimicrobial classes were classified as multidrug-resistant (MDR) as defined by a joint group of the European Centre for Disease prevention and Control and the Center for Disease Control and Prevention of the USA [6]. The frequency of antimicrobial resistance was considered as follows: rare: <0.1%; very low: 0.1–1.0%; low: >1–10%; moderate: >10–20%; high: >20–50%; very high: >50–70%; extremely high: >70%; according to the European Food Safety Authority and the European Centre for Disease Prevention and Control [7]. Data were tabulated using a spreadsheet (Microsoft Excel ^®^ 2019) and are presented as percentages with respective 95% confidence intervals. The Ethical Committee (“Comité de Ética para la Experimentación con Animales”) from the University of Antioquia on session number 133/2020 approved this study.

## 3. Results

### 3.1. Dog Isolates and Antimicrobial Susceptibility

**Figure 1:**
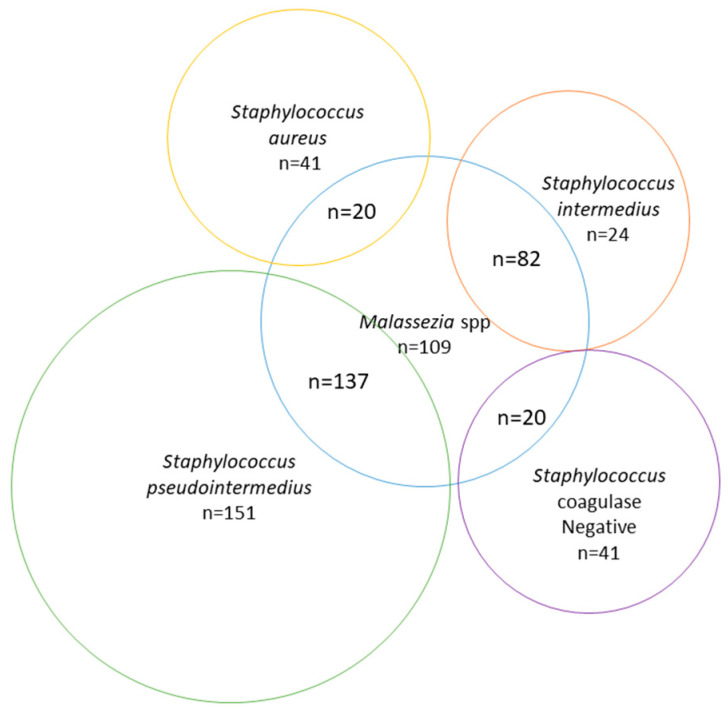
Number of infections by *Malassezia* spp. and *Staphylococcus* spp. isolated from skin and ear samples in dogs between 2016 and 2019.

In dogs, samples from ears (*n* = 667), wounds (*n* = 240), and urine (*n* = 226) represented the majority of the samples over the study period (Table 1). The most frequent bacterium isolated from clinical samples were *Staph.* coagulase-positive starting from wounds (65%), ears (64%), skin (48.9%), abscesses (38.9%), nasal cavity (35.3%), and eyes (33.3%). The exception was *Escherichia coli*, the species most common in urine samples (*n* = 46.5%). Wounds were more likely infected with *Staph.* coagulase-positive (65%) than *Staph*. coagulase-negative (10.4%); however, clinical samples from skin (i.e., superficial pyodermas) harbored the highest percentage of *Staph.* coagulase-negative (33.3%), closer to those of *Staph.* coagulase-positive ones (48.9%). When ear and skin infections were combined, there was bacterial and/or fungal growth in 605 samples (Figure 1). The number of mixed infections with *Malassezia pachydermatis* was 259, of pure *Malassezia pachydermatis* was 109 and of pure *Staphylococcus.* spp. was 257.

Antimicrobial susceptibilities for the 1316 bacteria isolates are presented in Table 2. *Staphylococcus* spp. (*n* = 771) accounted for the most common tested group, followed by *Enterobacteriaceae* (*n* = 183), *E. coli* (*n* = 163) and *Enterococcus* spp. (*n* = 90). Within the *Staphylococcus* group, the *Staph. aureus* species exhibited high resistance (20–50%) to ampicillin, cephalexin, enrofloxacin, gentamicin, trimetropim-sulfonamide, and extremely high resistance (75%) to tetracycline. However, all *Staph. aureus* isolates were susceptible to amikacin and amoxicillin-clavulanate. For the other *Staphylococcus* spp., most isolates had high resistance and only amoxicillin-clavulanate and cephalothin were able to reach levels of 90–100% susceptibility. For the *Enterobacteriaceae* group, there were high levels of resistance (23.6–47.8%) to 7 antimicrobials, and very high resistance (61.3%) to doxycycline (Table 2). Moderate (10–20%) to low (1–10%) levels of resistance were found in 3 antimicrobials and no resistance was found against amikacin and florfenicol. With regards to *E. coli*, resistance was also high (20–50%) for 11 antimicrobials, moderate (10–20%) for gentamicin and ampicillin-sulbactam, and zero for amikacin and florfenicol. The *Pseudomonas* spp. group had the most resistant isolates of bacteria studied. There were 8 antimicrobials against which the resistance would be considered extremely (>70%) high, while for cephalothin no isolates were susceptible (100% resistant). For this intrinsically resistant genera of bacteria, there was low (1–10%) to moderate (10–20%) resistance against amikacin, gentamicin and ciprofloxacin. Multi-drug resistance was observed in 18.27% (246/1346) of the dog isolates, ranging between 7% and 20% in all groups of bacteria except for *Pseudomonas* spp. which reached almost 50%.

### 3.2. Cat Isolates and Antimicrobial Susceptibility

In cats, there were only 94 samples submitted over the study period, and the majority were from wounds (*n* = 29), urine (*n* = 29), ears (*n* = 18), and nasal cavity (*n* = 10) (Table 3). The most common bacterium isolated was *Staphylococcus* coagulase-positive from ear (66.7%) and wounds (44.8%). *Escherichia coli* was the most common bacterium isolated from urine samples (31%), followed by different bacterial species of the *Enterobacteriaceae* family (24.1%).

A total of 110 antimicrobial susceptibilities are presented in Table 4. *Staphyloccocus* spp. was the largest tested group (*n* = 45), followed by the *Enterobacteriaceae* (*n* = 18) and *E. coli* (*n* = 13). Unlike in dogs, frequency of resistance in *Staphylococcus* spp. to the most common antimicrobials was low (6.7–9.1%) to moderate (11.1–20%), with the exception of all 5 isolates of *Staph. intermedius* in which resistance was high or very high to amoxicillin-clavulanate (40%), ampicillin (60%), cephalosporin (60%), and ciprofloxacin (50%). The *Enterobacteriaceae* family had high (30–40%) resistance to 6 antimicrobials, very high (50–70%) to ampicillin and cephalothin, and extremely high (>70%) to enrofloxacin. *E. coli* showed very high (30–40%) resistance against 7 antimicrobials, and only cephalothin exhibited 100% susceptibility. With regards to *Pseudomonas* spp., out of 8 isolates, there were very high resistance (50–70%) to three antimicrobials and only gentamicin managed to attain 100% susceptibility. Multi-drug resistance was observed in 22% (21/97) of the isolates.

## 4. Discussion

This is the first study to describe the prevalence of bacterial pathogens isolated from clinical samples submitted for culture and susceptibility testing from dogs and cats in Colombia. In general, most data on antimicrobial resistance in bacteria isolated from companion animals are usually reported for a specific site of infection or one type of bacteria. A couple of studies from other diagnostic laboratories provide information that can be contrasted with this study [1,2]. In the study by Awosile et al. [1], ear samples represented 46.5% of the case submissions, followed by urine (27.1%) and skin (7.8%). This study made major findings: (a) the level of resistance in all families of bacteria studied, except for *Streptococcus* spp., was estimated as high (20–50%) to at least 6 or more antimicrobials; (b) resistance to critically important antimicrobials used in human medicine (enrofloxacin, gentamicin) was high for most bacterial families, with a prevalence between 20 and 50%; of particular concern was resistance in zoonotic *Staph. aureus* for which 40% resistance was attained for enrofloxacin and gentamicin; (c) there were bacteria like *Pseudomonas* spp. for which there was an extremely high prevalence (>70%) of resistance against 10 antimicrobials, and only three antimicrobials exhibited low (1–10%) and moderate (10–20%) resistance; (d) for pathogenic *Pseudomonas* spp. and largely commensal *Enterococcus* spp. organisms, no antimicrobials reached 100% susceptibility; finally, (d) excluding *Pseudomonas* spp., multi-drug resistance was very high in all families of bacteria ranging between 10 and 20% of all isolates.

With regards to skin and ear infections and consistent with other studies, *Staphylococcus* coagulase-positive (CoPS), and in particular *Staphylococcus pseudointermedius*, was the most common organism isolated [8,9,10,11]. This was followed by *Staphylococcus* coagulase-negatives (CoNS), which other studies have identified mostly as *S. schleiferi*. In general, the species of *Staphylococci* that cause disease in people and pets are different, and the *S. intermedius* species entity (known as *Staph. pseudointermedius* after 2005) was for a long time useful to separate these coagulase-positive staphylococci from *Staph. aureus*, avoiding an epidemiologically important source of confusion. Yet, in one of the largest recent retrospective studies of 4972 canine *Staphylococcus* isolates diagnosed in a diagnostic laboratory, *Staph. pseudointermedius* was included within the *S. intermedius* group and still accounted for the largest group (68%, 3388/4972) among the *Staphylococcus* spp. infections [11]. In this last study, CoNS were the second largest group with 18.3% (907/4972), and *Staph. aureus* was the third group with only 5.8% (290/4972) of the isolates. Yet, antimicrobial resistance was slightly higher among CoNS and *Staph. aureus* with about 80% of the isolates resistant to at least one antimicrobial compared to 77% in the *Staph. pseudointermedius* group. By contrast, studies that have characterized the staphylococcal population structure and antimicrobial resistance profile in healthy dogs and cats have shown that it is the CoNS (with up to 22 different *Staph*. species), and not CoPS, that dominate the healthy skin and mucosal surfaces of dogs and cats [12,13]. For example, in the study by Schmidt et al. [12] in which *Staphylococci* were isolated from the nasal and perineal swabs of 73 Labrador Retrievers (*n* = 436 isolates), 95% (334) were CoNS and 47% (102) were CoPS. In addition, antimicrobial resistance was detected in the CoNS isolates from 38% of the dogs, despite the enrollment criteria in the study precluding prior antimicrobial treatment or admission to veterinary premises for at least 12 months. A study that also investigated the diversity of Staphylococcal species in healthy dogs and cats observed that CoNS were more prevalent in cats, with 86% (258/300) compared to 60% (172/284) in dogs [13]. They also showed that multi-drug resistant isolates occurred almost exclusively in CoNS for cats, but it was almost equally distributed between the CoNS and CoPS groups for dogs. Thus, the clinical significance of CoNS may not only be as opportunistic pathogens [8], but also as clear potential reservoirs of antimicrobial resistance genes that can be transferred to CoPS. Except for the skin, we found a much higher prevalence (6–10 fold) of CoPS than CoNS in samples from ears, wounds, nasal cavity, and abscesses; whether this implies a clinically relevant association is unknown, as the pathogenicity of CoNS has not been clearly established. It is clear that CoPS tends to predominate over CoNS when there are infections. A large retrospective study in France that compiled 7623 cases of dogs with otitis from 2012 to 2016 found a prevalence of *Staph. pseudointermedius* of 33% compared to a prevalence of 4.3% for all other *Staph* spp. combined [14]. In our study, multi-drug resistance was observed at about the same level (17–19%) for the CoPS isolates compared to 11% in the CoNS. Moreover, when examining antimicrobial susceptibility for *Staph pseudointermedius*, the following antimicrobials with their associated susceptibilities met the criteria for empirical treatment: amikacin (96.8%), amoxicilin-clavulanate (93.3%), ciprofloxacin (100%), and the third-generation cephalosporin cefoperozone (100%). Unfortunately, among the first-tier drugs that are recommended by the International Group of the International Society for Companion Animal Infectious Diseases (ISCAID) for the diagnosis and antimicrobial therapy of canine superficial bacterial folliculitis [15], only amoxicillin-clavulanate met the criteria for empirical treatment because cephalosporin and trimethoprim-sulphonamide showed unacceptable levels of resistance of 18% and 43%, respectively. In addition, resistance in *Staph. pseudointermedius* was equally prevalent across other types of *Staphylococcus* isolates, exemplified by all groups showing a high (20–50%) prevalence of resistance to five antimicrobials. These findings, together with all *Staphylococcus* showing MDR between 10 and 19%, imply a serious threat to the ability of veterinarians to treat patients for *Staphylococcus* spp. infections. However, except for the *Pseudomonas* spp. and *Enterococcus* spp. organisms, *Staphylococcus* spp. and other bacterial types had at least one antimicrobial that reached 100% susceptibility. Finally, among the *Staphylococcus* spp. organisms, 104/771 (13.4%) were *Staph. aureus* of which 19.7% were MDR, representing a public health concern as there is a risk of transfer of this resistant pathogen from pets to humans. In our small cat population, unlike the dog situation, the prevalence of resistance to a number of less antimicrobials tested was low (1–10%) for *Staph. pseudointermedius* and moderate (10–20%) for *Staph aureus* isolates, and both species showed 100% susceptibility against amikacin and gentamicin. In addition, a larger percentage of CoNS were generally isolated in cats compared to dogs, and *Pseudomonas* spp. was also isolated in more urinary samples than dogs.

One of the main risk factors that a large number of studies associate with resistance of *Staphylococcus pseudointermedius* in cases of pyoderma is the prior administration of antimicrobials, which in many cases is unnecessary as topical therapy is the best option, further emphasizing the need for prudent use of antimicrobials [16,17,18,19]. For example, in an epidemiological case study, the odds ratio of presenting methicillin-resistant *Staphylococcus pseudointermedius* versus methicillin-susceptible *Staph pseudointermedius* infection was nine times higher in dogs treated with systemic antimicrobials 30 days before being referred to the hospital [20]. In another study, apart from antimicrobial administration, a higher proportion of methicillin-resistant *Staphphylococcus pseudointermedius* was also seen in animals that received corticosteroids compared with those that did not [21]. A large proportion of these animals probably did not respond to the antimicrobial therapy because they had some underlying problems such as atopy, as shown in another study [22].

Consistent with other studies, *Escherichia coli* was the most frequently isolated uropathogen at 46.5%, followed by other *Enterobacteriaceae* with 34.1%. The order of frequency for other types of bacteria isolated after *E. coli* has been shown to fluctuate between studies, but typically include *Staphyloccus* spp., *Proteus* spp., and *Klebsiella* spp. (the latter in the *Enterobacteriaceae* family), *Enterococcus* spp., *Pseudomonas* spp., and *Streptococcus* spp. [23,24,25,26]. The prevalence of resistance in *E. coli* was high (20–50%) to 11 antimicrobials, with only amikacin and florfenicol showing no resistance. In an Australian report, 41% of the isolates were resistant to amoxicillin and 15% to trimethoprim-sulfamethoxazole [24]. Although it cannot be directly compared, in our study, resistance of *E. coli* against the combination of amoxicilin-clavulanate and trimetropim-sulphonamide was 33.3% and 22.4%, respectively. In a larger study across the United States that aimed to describe the susceptibility patterns of *E. coli* isolates from 2392 dogs and 780 cats (mostly from urinary tract infections), no isolates were susceptible to all drugs and all were resistant to 1 or more drugs [27]. As observed in the Australian study, resistance to amoxicillin-clavulanic acid was high at 40% but low for trimethoprim-sulfamethoxazole (7.9%). In fact, their recommendation was to avoid empirical treatment with drugs, which had more that 40% resistant isolates: doxycycline, cephalexin, ampicillin, and amoxicillin-clavulanic acid. The International Society for Companion Animal Infectious Disease Committee (ISCAID) have formulated guidelines with first-line antimicrobials for uncomplicated urinary tract infections, which include amoxicillin and trimethoprim-sulfonamide [28]. However, a change in empirical treatment guidelines is clearly necessary in light of the antimicrobial resistance to these first-line antimicrobials. In our case, it is clear that neither amoxicillin nor trimethoprim-sulfonamide can any longer be recommended as first-line drug options for urinary tract disease. The ISCAID considers a 10% increase in resistance within the population from baseline as a reasonable breakpoint to change the empirical drug choice. Thus, in light of multidrug resistance, the only drugs available for empirical treatment in urinary infections caused by *E. coli* would be amikacin or florfenicol. Both of these drugs were also shown to have 100% efficacy against the *Enterobacteriaceae* organisms, which represented the second largest group of uropathogens with 34.1% of the isolates. Unfortunately, in our cat population there was high (20–50%) resistance in 8/8 antimicrobials tested against *E. coli* and *Enterobacteriaceae* for cases of urinary tract infections.

For urinary tract infections (UTI), it is also common to find mixed cultures of gram-positive cocci with gram-negative rods [23,29]. For example, the study by Penna et al. [29] which sampled 348 dogs with UTI, found 146 (42%) mixed cultures, 132 (38%) gram-negative rods in pure culture, and 70 (20%) gram-positive cocci in pure culture. Of the 70 (20%) gram-positive bacteria, there were equal numbers of CoNS and CoPS and all isolates were resistant to at least 1 drug and 53 (75.7%) were multi-drug resistant. Their study not only highlighted the importance of CoNS in producing UTI, but also the high levels of resistance to amoxicillin (71.5%) and trimethoprim-sulfamethoxazole (67.2%). We also found 11.5% of *Staphylococcus* spp. in urine samples from dogs, but most (9.7%) were CoPS and a few (1.8%) CoNS. Moreover, in both CoPS and CoNS, there was low (1–10%) resistance against the combination of amoxicillin-clavulanate, but high resistance (40%) against trimetroprim-sulfonamide. A similar trend was observed in cats but the numbers were not sufficient to reach any conclusion. Thus, when confronted with a CoPS organism for a UTI infection in the absence of a susceptibility test, the first-line treatment would be the combination of amoxicillin-clavulanate.

In a retrospective epidemiological study in 1029 dogs classified with uncomplicated, complicated infections, and pyelonephritis, it was observed that those who had previously received antimicrobials (those who had complicated infections) had a higher number of multiresistant bacteria, particularly in *E. coli* and *Staphylococcus* spp. (36% versus 21%) [30]. In 21% of dogs with uncomplicated infections, that is, those that presented infections for the first time and had never been treated, there were multiresistant bacteria and no antimicrobial showed 100% susceptibility, 90% at the most. These findings are important because they imply that any dog, even if it has never received any antibiotics, may not initially respond to any of the antimicrobials administered.

Some of the limitations of this study were the small sample sizes for cats associated with each category, and consequently the low precision for each one. Furthermore, we could not investigate the association between previous antimicrobial use and resistance patterns. It is likely that animals included in this study were mostly those that had not responded to empirical treatments and were examined for a second or third time. It is obvious that knowledge of resistance patterns of bacteria to antimicrobial drugs requires constant vigilance. This study emphasizes the need for conducting bacterial cultures with species identification and antibacterial susceptibility in order to choose the appropriate drug for each case. There are ubiquitous bacteria like *Pseudomonas* spp. that can infect multiple anatomical sites, are intrinsically resistant to most antimicrobials, and for which antimicrobial testing should definitely be used to guide treatment selection.

## 5. Conclusions

This study has made it clear that multi-drug resistance is commonly present in bacteria isolated from animal infections in companion animals in Medellin. The data provide important baseline measurements for future surveillance and repeated surveys of this type are crucial for understanding trends of antimicrobial resistance in dogs and cats. It is likely that antimicrobial overuse in veterinary practices is creating selection pressure and surveys are underway to assess the rate of prescribing for the most common clinical conditions among veterinarians in the city of Medellin. Antimicrobial stewardship strategies and programs are already needed in Colombian companion animal practices. We propose the development of such a program at the teaching hospital of the University of Antioquia. It is undoubtedly time to initiate antimicrobial resistance monitoring programs in practice realms beyond just poultry.

## Figures and Tables

**Table 1:** Bacterial isolates from clinical samples from dogs submitted to the Diagnostic Laboratory of the Faculty of Agrarian Sciences, University of Antioquia (2016–2019).

Clinical Samples	*n*	*Enterobacteriaceae* ^a^	*Enterococcus* spp. ^b^	*Escherichia coli.*	*Pseudomonas* ^c^	*Staphylococcus* Coagulase Negative ^d^	*Staphylococcus* Coagulase Positive ^e^	*Streptococcus* spp.	Others ^f^
		% (95% CI)	% (95% CI)	% (95% CI)	% (95% CI)	% (95% CI)	% (95% CI)	% (95% CI)	% (95% CI)
Ear	667	9.1 (7.2–11.6)	7.3 (5.6–9.6)	3.3 (2.2–4.9)	7.9 (6.1–10.29	6.7 (5.0–8.9)	64.0 (60.3–67.6)	0.9 (0.4–1.9)	0.6 (0.2–1.5)
Wound	240	8.3 (5.5–12.5)	5.0 (2.9–8.5)	4.6 (2.5–8.0)	4.2 (2.3–7.5)	10.4 (7.1–14.9)	65.0 (58.8–70.7)	1.3 (0.4–3.6)	1.3 (0.40–3.6)
Urine	226	34.1 (28.2–40.5)	5.3 (3.1–9.0)	46.5 (40.1–52.9)	2.2 (0.9–5.0)	1.8 (0.7–4.5)	9.7 (6.5–14.3)	0.4 (0.1–2.5)	0 (0–1.7)
Skin	45	2.2 (0.4–11.6)	4.4 (1.2–14.8)	2.2 (0.4–11.6)	4.4 (1.2–14.8)	33.3 (21.3–47.9)	48.9 (35.0–63.0)	2.2 (0.4–11.6)	2.2 (0.4–11.6)
Nasal cavity	34	17.6 (8.3–33.5)	11.8 (4.7–26.6)	14.7 (6.4–30.1)	0 (0–10.2)	2.9 (0.52–14.9)	35.3 (21.5–52.1)	5.9 (1.6–19.1)	11.8 (4.7–26.6)
Abscess	18	5.6 (1.0–25.7)	22.2 (9.0–45.2)	22.2 (5.8–39.2)	11.1 (3.1–32.8)	0 (0–17.6)	38.9 (20.3–61.4)	0 (0–17.6)	5.6 (1.0–25.7)
Eyes	15	6.7 (1.19–29.8)	0 (0–20.4)	20.0 (7.0–45.2)	6.7 (1.2–29.8)	26.7 (10.9–51.9)	33.3 (15.2–58.3)	6.7 (1.2–29.8)	0 (0–20.4)
Surgical	11	0 (0–25.9)	9.1 (1.6–37.7)	27.3 (9.7–56.5)	27.3 (9.7–56.5)	18.2 (5.1–47.7)	18.2 (5.1–47.7)	0 (0–25.9)	0 (0–25.9)

^a^*Enterobacter* spp., *Klebsiella* spp., *Citrobacter* spp., *Proteus* spp., *Serratia* spp., *Shigella* spp., *Yersinia* spp., *Salmonella* spp. ^b^ *Enterococcus* spp., *Enterococcus faecalis*. ^c^ *Pseudomonas* spp., *Flavimonas* spp., *Acinetobacter* spp. ^d^ *Staphylococcus saprophyticus*, *Staphylococcus epidermidis*, *Staphylococcus haemolyticus*. ^e^ *Staphylococcus aureus*, *Staphylococcus intermedius*, *Staphylococcus pseudointermedius*. ^f^ *Corynebacterium* spp., *Gardnerella vaginalis*, *Stenotrophomonas maltophilia*, *Morganella morganii*, *Gemella palaticanis*, *Chromobacterium violaceum*, *Sphingomonas paucimobilis*, *Pasteurella multocida*.

**Table 2:** Antibacterial susceptibilities in bacteria isolated from clinical samples of dogs (2016–2019).

Bacteria	*n*	% Susceptible																				MDR (%)
		AMK	AMC	AMP	CEX	ENO	GEN	TMS	DOX	TET	CIP	FLO	PEN	AMPS	CET	RIF	CEF	NEO	CHL	ERY	CFP	
*Enterobacteriaceae*	183	100	76.7	61.8	60.9	64.4	89	83.9	38.7	52.2	84.4	100	-	75	-	-	56.9	83.3	-	-	-	18.6
*Enterococcus* spp.	90	66.7	89.7	84.9	28.6	24.7	64.7	36.4	38.3	50.9	66.7	96	88.2	88.2	-	-	42.9	-	80	20	-	20.0
*Escherichia coli*	163	100	66.7	67.7	72.3	69.2	89.8	77.6	68.7	64.5	75	100	-	83.3	57.4	-	70.7	66.7	-	-	-	17.2
Other *	14	87.5	90	91.7	100	90.9	88.9	83.3	-	-	-	-	-	-	-	-	-	-	-	-	-	7.1
*Pseudomonas* spp.	79	93.9	14.7	16.2	8.3	31.8	86.7	21.4	17.2	12.5	91.4	-	-	17.2	-	-	0%	33.3	-	-	-	49.4
*Staphylococcus coagulase Negative*	101	78.2	96.9	77.4	83.5	82.7	73.3	65.3	100	38.5	66.7	-	-	50	-	-	100	71.4	-	-	-	10.9
*Staphylococus* *aureus*	104	100	100	75.5	74.7	59.8	59.8	62.3	91.7	25	92.3	-	-	-	-	-	-	-	-	-	-	19.2
*Staphylococus* *intermedius*	160	-	84.4	74.7	73.3	58.6	80	60	70	-	85.5	-	-	-	-	100	96.6	75.6	-	-	-	16.9
*Staphylococos* *pseudointermedius*	406	96.8	93.3	77.6	81.6	82.3	63.9	57	60.9	62.8	100	-	-	76	-	-	86.7	-	-	-	100	16.7
Streptococcus spp.	16	42.9	91.7	86.7	80	58.3	75	100	-	25	-	-	-	-	-	-	100	-	-	-	-	12.5

AMK: Amikacina, AMC: Amoxicillin-clavulanate, AMP: Ampicillin, CEX: Cephalexin, ENO: Enrofloxacin, GEN: Gentamicin, TMS: Trimethoprim-sulfadiazine, DOX: doxycyclin, TET: Tetracycline, CIP: Ciprofloxacina, FLO: florfenicol, PEN: Penicillin, AMPS: Ampicillin sulbactam, CET: ceftiofur, RIF: Rifampicin, CEF: Cephalothin, NEO: Neomycin, CHL: Chloramphenicol, ERY: Erythromycin, CFP: Cefoperazone, MDR: Multi-drug resistant. (-) not determined. *Other: *Corynebacterium* spp., *Gardnerella vaginalis, Stenotrophomonas maltophilia, Morganella morganii, Gemella palaticanis, Chromobacterium violaceum, Sphingomonas paucimobilis, Pasteurella multocida.* **Interpretation of colors**: DARK BLUE: 0.1–1% very low resistance, BLUE: >1–10% low resistance, PURPLE: >10–20% moderate resistance, RED: >20–50% high resistance, Light Green: >50–70% very high resistance, Dark Green: >70% extremely high resistance.

**Table 3:** Bacterial isolates from clinical samples from cats submitted to the Diagnostic Laboratory of the Faculty of Agrarian Sciences, University of Antioquia (2016–2019).

Clinical Samples	*n*	*Enterobacteriaceae* ^a^	*Enterococcus* spp. ^b^	*Escherichia coli.*	*Pseudomonas* ^c^	*Staphylococcus* Coagulase Negative ^d^	*Staphylococcus* Coagulase Positive ^e^	*Streptococcus* spp. ^f^	Others
		% (95% CI)	% (95% CI)	% (95% CI)	% (95% CI)	% (95% CI)	% (95% CI)	% (95% CI)	% (95% CI)
Wound	29	17.2 (7.6–34.5)	10.3 (3.6–26.4)	6.9 (1.9–21.9)	6.9 (1.9–21.9)	6.9 (1.9–22.0)	44.8 (28.4–62.5)	3.4 (0.6–17.2)	3.4 (0.6–17.2)
Urine	29	24.1 (12.2–42.1)	6.9 (1.9–21.9)	31 (17.3–49.2)	13.8 (5.5–30.6)	10.3 (3.6–26.4)	13.8 (5.5–30.6)	0 (0–11.7)	0 (0–11.7)
Ear	18	5.6 (1–25.7)	5.6 (0.9–25.8)	0 (0–17.6)	5.6 (0.9–25.7)	16.7 (5.8–39.2)	66.7 (43.7–83.7)	0 (0–17.6)	0 (0–17.6)
Nasal cavity	10	20 (5.7–50.9)	10 (1.8–40.4)	0 (0–27.7)	0 (0–27.7)	30 (10.8–60.3)	30 (10.8–60.3)	0 (0–27.7)	10 (1.8–40.4)
Skin	3	33.3 (6.14–79.23)	33.3 (6.1–79.2)	33.3 (6.1–79.2)	0 (0–56.1)	0 (0–56.1)	0 (0–56.1)	0 (0–56.1)	0 (0–56.1)
Surgical	2	0 (0–65.8)	50 (9.5–90.5)	0 (0–65.8)	0 (0–65.8)	0 (0–65.8)	50 (9.5–90.5)	0 (0–65.8)	0 (0–65.8)
Abscess	2	0 (0–65.8)	0 (0–65.7)	0 (0–65.8)	0 (0–65.8)	0 (0–65.8)	50 (9.5–90.5)	50 (9.5–90.5)	0 (0–65.8)
Eyes	1	0 (0–79.3)	100 (20.6–100)	0 (0–79.3)	0 (0–79.3)	0 (0–79.3)	0 (0–79.3)	0 (0–79.3)	0 (0–79.3)

^a^*Enterobacter* spp., *Klebsiella* spp., *Citrobacter* spp., *Proteus* spp., *Serratia* spp., *Shigella* spp., *Yersinia* spp., *Salmonella* spp. ^b^ *Enterococcus* spp., *Enterococcus faecalis*. ^c^ *Pseudomonas* spp., *Flavimonas* spp., *Acinetobacter* spp. ^d^ *Staphylococcus saprophyticus*, *Staphylococcus epidermidis*, *Staphylococcus haemolyticus*. ^e^ *Staphylococcus aureus*, *Staphylococcus intermedius*, *Staphylococcus pseudointermedius*. ^f^ *Corynebacterium* spp., *Gardnerella vaginalis*, *Stenotrophomonas maltophilia*, *Morganella morganii*, *Gemella palaticanis*, *Chromobacterium violaceum*, *Sphingomonas paucimobilis*, *Pasteurella multocida*.

**Table 4:** Antibacterial susceptibilities in bacteria isolated from clinical samples of cats (2016–2019).

Bacteria	*n*	% Susceptible												MDR (%)
		AMK	AMC	AMP	CEX	ENO	GEN	TMS	DOX	TET	CIP	FLO	CEF	
*Enterobacteriaceae*	18	-	72.2	46.2	66.7	26.7	66.7	63.6	61.5	80	66.7	-	50	33.3
*Staphylococus* *aureus*	16	100	85.7	83.3	64.3	93.3	100	88.9	-	-	92.3	-	-	6.3
*Escherichia coli*	13	-	66.7	54.5	72.3	63.6	70	60	62.5	64.5	-	-	100	30.8
Others	13	-	-	-	-	-	-	-	-	-	-	-	-	7.7
*Staphylococos* *pseudointermedius*	13	100	100	91.7	100	92.3	100	57	-	-	-	-	-	0.0
*Enterococcus* spp.	11	-	63.6	75	-	37.5	-	25	25	42.9	-	87.5	-	18.2
*Staphylococcus coagulase Negative*	11	45.5	100	90.9	80	81.7	85.7	100	-	-	-	-	-	18.2
*Pseudomonas* spp.	8	-	-	-	-	50	100	50	50	-	-	-	-	37.5
*Staphylococus* *intermedius*	5	-	60	40	40	-	-	-	-	-	50	-	-	40.0
*Streptococcus* spp.	2	-	-	-	-	-	-	-	-	-	-	-	-	0.0

AMK: Amikacina, AMC: Amoxicillin-clavulanate, AMP: Ampicillin, CEX: Cephalexin, ENO: Enrofloxacin, GEN: Gentamicin, TMS: Trimethoprim-sulfadiazine, DOX: doxycyclin, TET: Tetracycline, CIP: Ciprofloxacina, FLO: florfenicol, CEF: Cephalothin. MDR: Multi-drug resistant. Other: *Corynebacterium* spp., *Gardnerella vaginalis, Stenotrophomonas maltophilia, Morganella morganii, Gemella palaticanis, Chromobacterium violaceum, Sphingomonas paucimobilis, Pasteurella multocida*. (-) not determined. **Interpretation of colors**: DARK BLUE: 0.1–1% very low resistance, BLUE: >1–10% low resistance, PURPLE: >10–20% moderate resistance, RED: >20–50% high resistance, Light Green: >50–70% very high resistance, Dark Green: >70% extremely high resistance.
